# Delivery of microRNA-33 Antagomirs by Mesoporous Silica Nanoparticles to Ameliorate Lipid Metabolic Disorders

**DOI:** 10.3389/fphar.2020.00921

**Published:** 2020-08-05

**Authors:** Yaoye Tao, Shengjun Xu, Jianguo Wang, Li Xu, Chenzhi Zhang, Kangchen Chen, Zhengxing Lian, Junbin Zhou, Haiyang Xie, Shusen Zheng, Xiao Xu

**Affiliations:** ^1^ Department of Hepatobiliary and Pancreatic Surgery, First Affiliated Hospital, Zhejiang University School of Medicine, Hangzhou, China; ^2^ NHC Key Lab of Combined Multi-Organ Transplantation, Hangzhou, China

**Keywords:** mesoporous silica nanoparticles, lipid metabolic disorder, non-alcoholic fatty liver disease, dyslipidemia, miR-33

## Abstract

Lipid metabolic disorders have become a major global public health concern. Fatty liver and dyslipidemia are major manifestations of these disorders. Recently, MicroRNA-33 (miR-33), a post-transcriptional regulator of genes involved in cholesterol efflux and fatty acid oxidation, has been considered as a good therapeutic target for these disorders. However, the traditional methods of gene therapy impede their further clinical transformation into a mature treatment system. To counter this problem, in this study we used mesoporous silica nanoparticles (MSNs) as nanocarriers to deliver miR-33 antagomirs developing nanocomposites miR-MSNs. We observed that the hepatocellular uptake of miR-33 antagomirs increased by ∼5 times when they were delivered using miR-MSNs. The regulation effects of miR-MSNs on miR-33 and several genes involved in lipid metabolism were confirmed in L02 cells. In a high-fat diet fed mice, miR-33 intervention *via* miR-MSNs lowered the serum triglyceride levels remarkably by 18.9% and reduced hepatic steatosis. Thus, our results provide a proof-of-concept for a potential strategy to ameliorate lipid metabolic disorders.

## Introduction

Lipid metabolic disorders are key contributors to many illnesses, such as cardiovascular diseases (CVDs) and type 2 diabetes mellitus; they are now considered a serious threat to human health (Zhou et al., 2019). Nonalcoholic fatty liver disease (NAFLD), a manifestation of lipid metabolic disorders, is the most common cause of chronic liver disease worldwide, affecting one-third of the total adult population in the world (Younossi et al., 2016; Younossi et al., 2018). One of the primary pathological signs of NAFLD is an excess of triglyceride accumulation in hepatocytes. This condition is called steatosis and is related to dyslipidemia (Woods et al., 2015). Although simple steatosis does not have serious implications in the beginning, NAFLD, particularly its histological phenotype non-alcoholic steatohepatitis, can potentially result in cirrhosis, end-stage liver disease, and hepatocellular carcinoma (Buzzetti et al., 2016). There are currently no approved pharmacological therapies for NAFLD and thus it has become the second leading cause for liver transplantation (LT) in the United States (Wong et al., 2015; Rotman and Sanyal, 2017). However, 30%–60% of patients who undergo LT due to NAFLD relapse in less than five years. In addition, 20%–30% of patients undergoing LT for other liver diseases also suffer from NAFLD and 40%–66% of LT patients suffer from dyslipidemia (Zheng and Wang, 2015; Pais et al., 2016). Due to the high prevalence of lipid metabolic disorders after transplantation, CVDs have become the leading cause of non-hepatic mortality in LT recipients, accounting for 11% of deaths annually. It has been found that the incidence of CVD events after LT increases over time (Lee et al., 2017; Fatourou and Tsochatzis, 2019). Therefore, new treatment modalities for lipid metabolic disorders, especially NAFLD, are urgently needed.

MicroRNA-33 (miR-33) is a post-transcriptional regulator of genes involved in cholesterol efflux and fatty acid oxidation; it is encoded within the introns of sterol regulatory element–binding proteins 1 and 2 (SREBF-1 and SREBF-2), which are key transcriptional modulators of lipid metabolism and are abundant in the liver. MiR-33 can suppress the expression of cholesterol efflux proteins, ATP-binding cassette A1 (ABCA1), and ATP-binding cassette subfamily G member 1 (ABCG1), and genes involved in fatty acid oxidation, carnitine palmitoyltransferase 1A (CPT1A), and carnitine O-octanoyltransferase (CROT) (Marquart et al., 2010; Najafi-Shoushtari et al., 2010; Rayner et al., 2010; Dávalos et al., 2011; Karunakaran et al., 2015). Moreover, miR-33 can promote the expression of SREBF-1, which is involved in the synthesis of fatty acids. Therefore, gene therapy based on miR-33, which delivers genetic material to genes for targeted therapy, has attracted the attention of researchers (Kulkarni et al., 2018). Studies on mice and nonhuman primates suggested that an miR-33 blockade results in changes in the expression of hepatic genes involved in lipid metabolism; further, it ameliorates dyslipidemia that is induced by a high-fat diet (HFD) (Rayner et al., 2011a; Rayner et al., 2011b). However, the traditional methods used in these studies yielded a limited delivery efficacy *in vivo* and inhibited further applications in clinic (He et al., 2018). One of the problems associated with the low efficiency of the traditional methods is that they require high doses of agents, with mice needing 10 mg/kg of anti-miR-33 oligonucleotides per week (Rayner et al., 2011b).

In recent times, safe, smart, and highly efficient nanomaterials have been gaining importance for their roles in treating different diseases, including NAFLD and dyslipidemia (Wang et al., 2016; Cao et al., 2018; Wan et al., 2018; Lee et al., 2019). Moreover, nanocarriers are promising carriers for gene delivery (Canup et al., 2017; Wang et al., 2018; Zai et al., 2019). Among the various types of nanoparticles (NPs) being used, mesoporous silica nanoparticles (MSNs) are promising vectors, owing to their unique characteristics of a tailored structure, large surface area, high agent-loading volume, abundant chemistry functionality, and acceptable biocompatibility (Tang et al., 2012; Zhou et al., 2018; Tao et al., 2020). Owing to these distinctive features, MSNs can serve as multifunctional and efficient platforms for gene delivery (Keasberry et al., 2017; Lin et al., 2018).

In this study, we attempted to deliver miR-33 antagomirs using MSNs to treat NAFLD and dyslipidemia. Aminated MSNs, such as MSNs-NH_2_, possess a net positive charge and form a stable complex with electronegative nucleic acid miR-33 antagomirs, named miR-MSNs. Our results suggest that the designed miR-MSNs can effectively facilitate hepatocellular uptake of miR-33 antagomirs. In vivo studies showed that intervention with miR-MSNs for a period of 2 weeks, which extended the retention time of miR-33 antagomirs to maximize the effect of therapy, can significantly decrease lipid deposition in liver, thus ameliorating metabolic disorders in HFD-fed mice. Therefore, this study provides a novel strategy for efficiently combating lipid metabolic disorder using MSNs-based nanoplatforms.

## Materials and Methods

### Materials

MiR-33 antagomirs and Cy5-labeled miR-33 antagomirs (Cy5-antagomirs) were purchased from GenePharma (Shanghai, China). The sequence of miR-33 antagomirs for mice is UGCAAUGCAACUACAAUGCAC; this sequence is the same for mice and humans. MSNs-NH_2_ were purchased from So-Fe Biomedical (Shanghai, China). Dulbecco’s Modified Eagles’s Medium (DMEM) and Trypsin-EDTA (0.25%) were purchased from Thermo Fisher Scientific (Waltham, USA). Fetal bovine serum (FBS) was purchased from Wisent (Montreal, Canada). Lipofectamine 3000 (lipo3000) was purchased from Invitrogen (Grand Island, NY). Oleic acid (OA) was purchased from Sigma-Aldrich (St Louis, MO, USA). Lovastatin was obtained from Glpbio (Montclair, USA). All other solvents were of an analytical grade. Fresh double-distilled water was used in all the experiments.

### Preparation of miR-MSNs

To prepare miR-MSNs, MSNs-NH_2_ and miR-33 antagomirs were mixed at a ratio of 100:1 (w/w) in RNase-free H_2_O. The samples were incubated at room temperature for 60 min to ensure NPs’ formation. NPs morphology was observed using a transmission electron microscope (TEM, Spirit 120kV, China). In addition, particle-size distribution and zeta (ζ) potential (ZP) were measured on a Malvern Nano-ZS90 instrument (Malvern, U.K.). Elemental distribution analysis by energy dispersive spectrum (EDS) was obtained on a field emission scanning electron microscope (Nova Nano 450, FEI Company, Czech).

### Cell Culture, *In Vitro* Transfection Studies and Co-Culture With Fatty Acids

Human hepatocyte L02 cells, hepatic fibroblasts LX02 cells, and mouse mononuclear macrophage leukemia RAW264.7 cells were purchased from the Cell Bank of China Science (Shanghai, China). These cells were cultured in DMEM supplemented with 10% FBS. For the transfection of miR-33 antagomirs, L02 cells were plated at a density of 2 × 10^5^ cells per well in six-well plates. Cells incubated overnight were transfected with 100 nM of miR-33 antagomirs using lipo3000 or miR-MSNs. Cells were then treated with OA (50 µM) for 72h and harvested for further detection.

### Agarose Gel Electrophoresis

Agarose gel electrophoresis was conducted to evaluate the loading of miR-33 antagomirs in MSNs. The antagomirs or miR-MSNs (antagomirs dose was 100 pmol) were separated on 2% agarose gels containing Super Gelred (US Everbright, Jiangsu, China) and the corresponding images were obtained using a UV transilluminator system (Life Science Technologies, USA).

### Cytotoxicity Study

Cells were seeded on 96-well plates at a density of 5 × 10^3^ cells/well and incubated overnight. Later, they were treated with MSNs-NH_2_ and miR-MSNs (125, 62.5, 31.25, 15.625, 7.8125, and 3.90625 µg/mL) for 72 h and the number of viable cells were measured using CCK-8 kits (MedChemExpress, USA) according to the protocol included in the user manual. Cell viability in each group was expressed as a percentage of the viability of untreated control cells.

### Hemolysis Assay

700 µL of MSNs-NH_2_ and miR-MSNs (200, 100, 50, 25, 12.5, and 6.25 µg/ml) solution was added to 700 µL of 2% w/v red blood cell (RBC) suspension and incubated at 37 °C for 1.5 h. Later, the mixtures were centrifuged and the supernatant was transferred to a 96-well plate (150 µL per well); hemoglobin release in these wells was measured by spectrophotometry as absorbance (A) at 540 nm. Deionized water and phosphate-buffered saline (PBS) were used as positive and negative controls, respectively. The extent of hemolysis was calculated as (A_sample_ − A_PBS_)/(A_water_ − A_PBS_) × 100%.

### *In Vitro* Cellular Uptake

The cellular uptake of Cy5-antagomirs of different formulations was examined using confocal laser scanning microscopy (CLSM) and flow cytometry analysis. Five groups were analyzed in this study: control, antagomirs (100 nM Cy5-antagomirs), lipo3000+antagomirs (100 nM Cy5-antagomirs), miR-MSNs (low) (50 nM Cy5-antagomirs), and miR-MSNs (high) (100 nM Cy5-antagomirs) groups. L02 cells were initially seeded on 3a 5 mm glass-bottom cell-culture dish (Thermo Scientific Nunc, USA) at a density of 4 × 10^5^ cells dish^−1^ and incubated for 24 h. Later, Cy5-antagomirs containing different formulations (stated above) were added to the cells, which were then incubated for three more hours. After incubation, the cells were washed thrice with PBS and immediately visualized under CLSM (Olympus Fluoview FV-3000, Japan) with red channel (Cy5-antagomirs) excitation at 640 nm. ImageJ was used to quantify fluorescence intensity. In flow-cytometry experiments, L02 cells were grown on 6-well plates (3 × 10^5^ cells per well) and exposed to Cy5-antagomirs with different formulations for 3h. The free Cy5-antagomirs containing formulations were removed by PBS washing. Subsequently, cells were harvested and centrifuged at 300 g for 5 min. Finally, cells were suspended in PBS and immediately analyzed by ACEA NovoCyteTM (ACEA Biosciences, USA).

### RT-PCR Analysis

Total RNA of L02 cells was extracted using NucleoZOL reagent (Macherey-Nagel, Germany) and then reverse transcribed into cDNA using a reverse transcription reagent Kit (Guangzhou Geneseed Biotech, China). Real-time PCR analysis was conducted with ChamQ™ SYBR qPCR Master Mix (Vazyme Biotech, China).

### Western Blot Analysis

Cell lysates were prepared using RIPA lysis buffer (Hangzhou Fude Biological Technology, China) containing 1 × protease cocktail inhibitor (Sigma-Aldrich, USA). Samples of the proteins were run on 8%–12% SDS-PAGE gels for immunoblotting. The primary antibodies used were SREBF1 (1:1000, Proteintech, USA), ABCA1 (1:1000, Abcam, UK), and β-actin (1:5000, Genscript, China). Matching horseradish peroxidase (HRP) conjugated secondary antibodies (1:5000, Genscript, China) were used to evaluate protein expression and the results were analyzed on a ChemiDoc Touch Imaging System (Bio-Rad Laboratories, USA).

### Cell Staining With Oil Red O and Lipid Measurement

For Oil Red O staining, L02 cells were washed twice with PBS, then fixed with 10% formalin for 30 min, and stained with Oil Red for 1 h before microscopic observation. For lipid measurement, the cells were ultrasonically broken using an ultrasonic cell disruptor (Sonics, USA). The levels of total cholesterol (TC) and triglyceride (TG) in the cells were measured using assay kits (Nanjing Jiancheng Bioengineering, China) according to the manufacturer’s protocol.

### Animals and Treatment

All animal studies were executed with the approval of the Institutional Animal Care and Use Committee of Zhejiang University. Male C57BL/6J (4 weeks) mice were purchased from the Shanghai Experimental Animal Center, Chinese Academy of Sciences. The mice were housed in a (20 ± 1) °C temperature-controlled room with a 12 h light/dark cycle and free access to food and water. To induce NAFLD and dyslipidemia, the mice were fed with HFD (60% kcal fats, 20% kcal carbohydrates, and 20% kcal proteins, n = 30). After 2 weeks, six mice were sacrificed, and their livers were harvested. The rest of the HFD mice were randomly divided into four groups (n=6), a model control group (MC group, untreated), a lovastatin group (5 mg/kg lovastatin, twice a week), a low dose miR-MSN group (25 mg/kg miR-MSNs, twice a week), and a high dose miR-MSNs group (50 mg/kg miR-MSNs, twice a week), and treated for 2 more weeks. During the study, the animals were weighed twice a week and their plasma was obtained on Day 0, 14, 21, and 28 from their eye-socket veins. At the end of the experimental period, liver, heart, spleen, lung, and kidney tissues were harvested from these mice. The fresh tissues were divided into two portions, half of which were immediately frozen in liquid nitrogen and stored at −80°C, and the remaining of which were fixed with 4% paraformaldehyde for histological analysis.

### *In Vivo* Biodistribution Studies

For *in vivo* imaging, the mice were randomly divided into two groups (n = 12 in each group) and injected with a single dose of Cy5-antagomirs or miR-MSNs (0.25 mg/kg equivalent Cy5-antagomirs) *via* the tail vein. At each predetermined time point, three mice in each group were sacrificed and major organs (heart, liver, spleen, lung, and kidney) were collected for *ex vivo* imaging. Fluorescent Cy5-antagomirs was used for measuring by an *in vivo* imaging system (IVIS) (Clairvivo OPT, SHIMADZU Corporation, Japan).

### Histological Staining

Mice-tissue samples embedded in paraffin wax were sectioned into 4 μm thick samples in the maximum cut area and stained with hematoxylin and eosin (HE) for microscopic observation. Hepatic fat accumulation was evaluated by Oil Red O staining. Liver-tissue samples stored at −80°C were sectioned and stained with 0.1% Oil Red to detect lipid droplets.

### Serum Biochemical Analysis

Plasma levels corresponding to TC, TG, alanine aminotransferase (ALT), aspartate aminotransferase (AST), creatinine (CRE), and blood urea nitrogen (BUN) were measured using commercially available kits (Changchun Huili Biotech, China) on a Chemray 240 automatic biochemical analyzer (Rayto Life and Analytical Sciences, China), according to the manufacturer’s protocol.

### Statistical Analysis

All the observed analytical results are reported as mean ± standard deviation (SD). Statistical analysis was conducted on SPSS 23.0 (two-sided student’s t-test or one-way ANOVA *post hoc*). *p < 0.05, **p < 0.01, and ***p <0.001 were considered to be statistically significant.

## Results and Discussion

### Preparation and Characterization of miR-MSNs

**Scheme 1:**
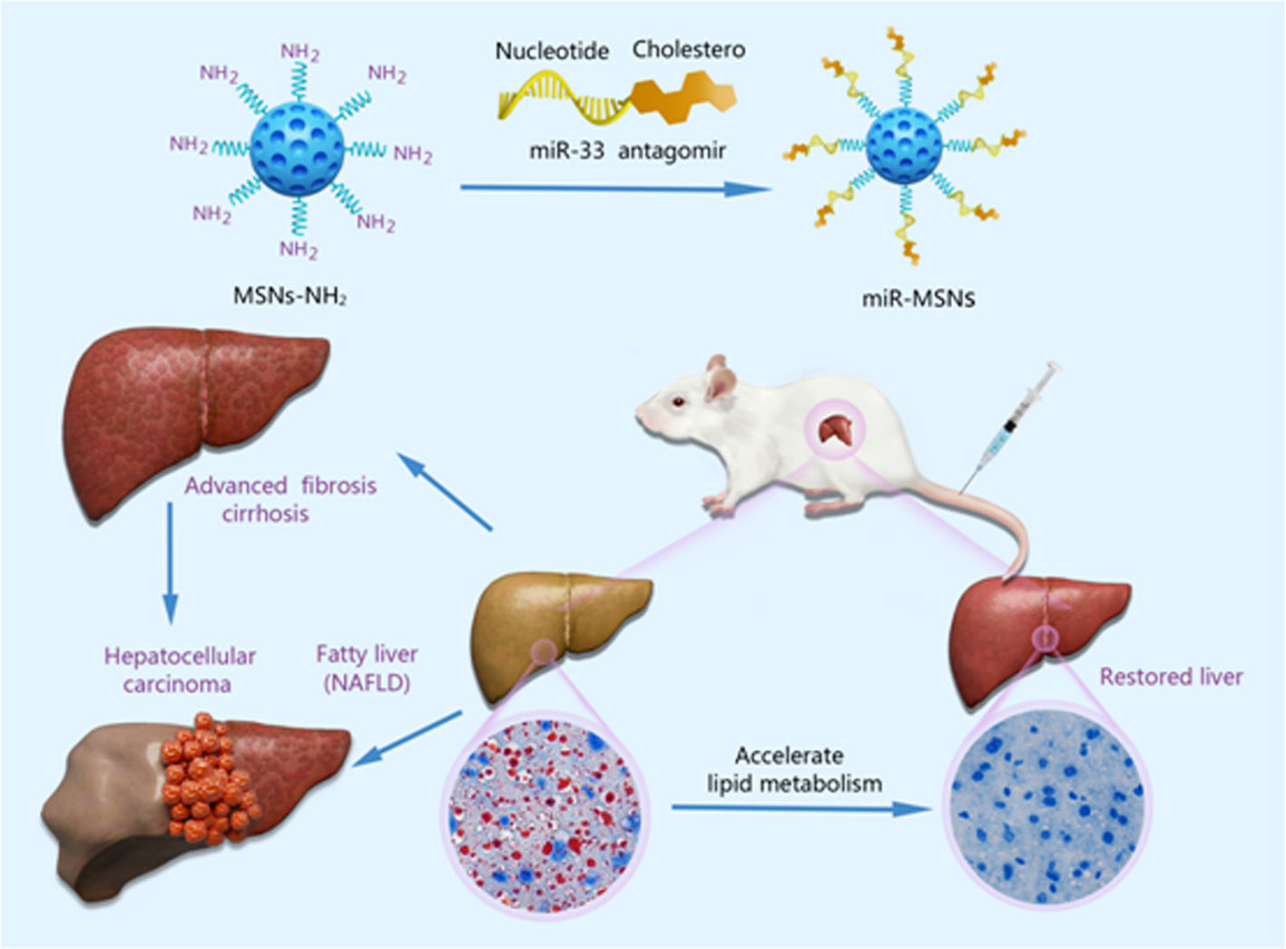
Schematic illustration of the miR-MSNs targeting the miR-33 to treat NAFLD.

**Figure 1:**
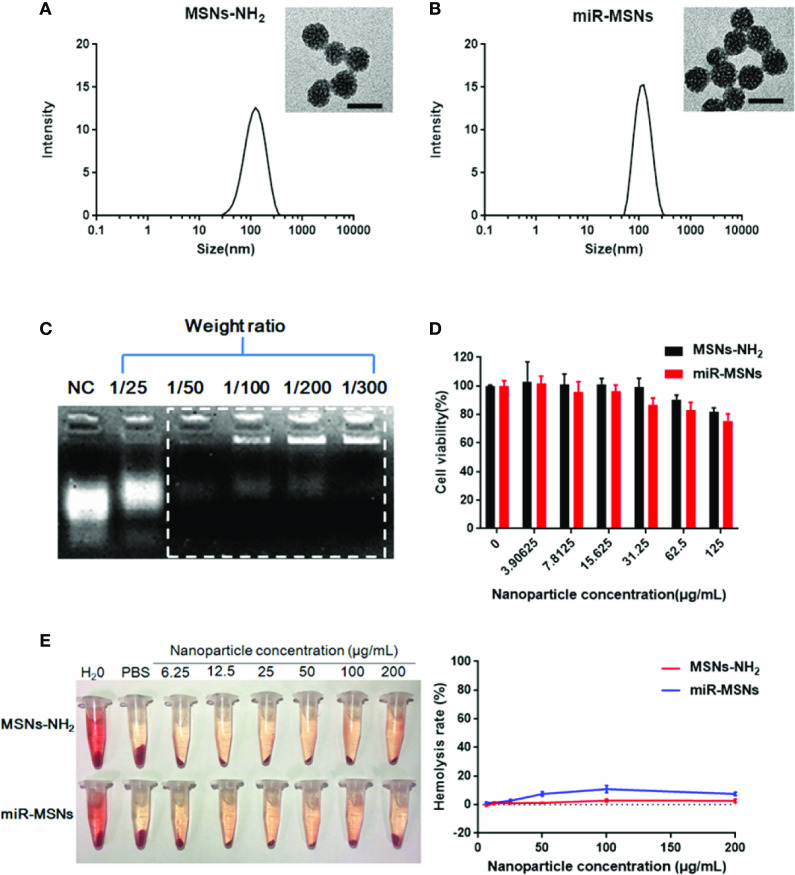
**(A)** Hydrodynamic diameter of MSNs-NH_2_ and the corresponding TEM image. Scale bar = 50 nm. **(B)** Hydrodynamic diameter of miR-MSNs and the corresponding TEM image. Scale bar = 50 nm. **(C)** Agarose gel electrophoresis of miR-33 antagomirs bonded with MSNs-NH_2_ at different weight ratios. **(D)** Cytotoxicity of MSNs-NH_2_ and miR-MSNs *via* CCK-8 assays. n=3. **(E)** Hemolysis assay and images of RBCs incubated with MSNs-NH_2_ and miR-MSNs. n=3.

Our strategy for the design and synthesis of miR-MSNs is illustrated in **Scheme 1**. Briefly, miR-33 antagomirs were incorporated into MSNs-NH_2_ by constant stirring. The particle sizes and size distributions of MSNs-NH_2_ and miR-MSNs are detailed in **Table 1**; as shown in the table, the fabricated NPs exhibited hydrodynamic diameters of 110−130 nm, which means that they can theoretically escape the reticulo-endothelial system in blood circulation (Tang et al., 2012). MSNs-NH_2_ exhibited an average size of (108.8 ± 0.6) nm, while miR-MSNs were (131.1 ± 5.4) nm in size (**Figures 1A, B**). Compared to MSNs-NH_2_, miR-MSNs were ∼20 nm larger in size and this difference may be regarded as evidence of the successful loading of miR-33 antagomirs in the latter. The polydispersity index (PDI) of these NPs was in the range of 0.154−0.227, which is acceptable. The ZP of MSNs-NH_2_ was (24.8 ± 1.9) mV, which is suitable for loading miR-33 antagomirs (Keasberry et al., 2017). The low ZP of miR-MSNs is due to the incorporation of the antagomirs, which is consistent with previous reports (Xue et al., 2017; Lin et al., 2018). The morphologies of the MSNs-NH_2_ and miR-MSNs were evaluated by TEM (**Figures 1A, B**). All the NPs exhibited a uniform mesoporous structure and monodispersed spherical shape. The capability of MSNs-NH_2_ for miR-33 antagomirs loading could be confirmed by agarose gel electrophoresis using untreated miR-33 antagomirs as negative control (NC). There remained a considerable amount of unbonded miR-33 antagomirs at a MSNs-NH_2_: miR-33 antagomirs ratio of 25:1 (w/w). When this ratio was greater than 50:1 (w/w), the NPs exhibited an adequate loading capacity (**Figure 1C**). Based on these results, we chose a 100:1 weight ratio for the follow-up experiment. EDS results showed that the distributions of Si and O are similar between MSNs-NH_2_ and miR-MSNs, while miR-MSNs contain more P elements than MSNs-NH_2_ (**Supplementary Figure 1**). The increase of P elements probably comes from miR-33 antagomirs incorporated in MSNs-NH_2_, reflecting successful formation of miR-MSNs. The cytotoxicity of MSNs-NH_2_ and miR-MSNs with respect to L02 cells was evaluated. The viability of L02 cells was greater than 82% after incubation with 125 µg/mL of MSNs-NH_2_ and miR-MSNs for 72 h (**Figure 1D**). Similarly, low cytotoxicity of MSNs-NH_2_ and miR-MSNs was also found in LX02 and RAW274.6 cells (**Supplementary Figure 2**). **Figure 1E** shows that hemolytic activity of MSNs-NH_2_ and miR-MSNs with respect to RBCs was negligible. The low cytotoxicity and hemolytic activity of these NPs can be mainly ascribed to their well-ordered mesoporous structure and suitable particle size (Lin and Haynes, 2010). These results clearly indicate that MSNs-NH_2_ and miR-MSNs are biocompatible and show no significant toxicity toward tissues and cells.

**Table 1:** Characterization of the prepared NPs.

NP	Size (nm)	PDI	ZP(mV)
MSNs-NH_2_	108.8 ± 0.6	0.154 ± 0.013	24.8 ± 1.9
miR-MSNs	131.1 ± 5.4	0.227 ± 0.009	17.4 ± 0.7

### *In Vitro* Cellular Uptake

**Figure 2:**
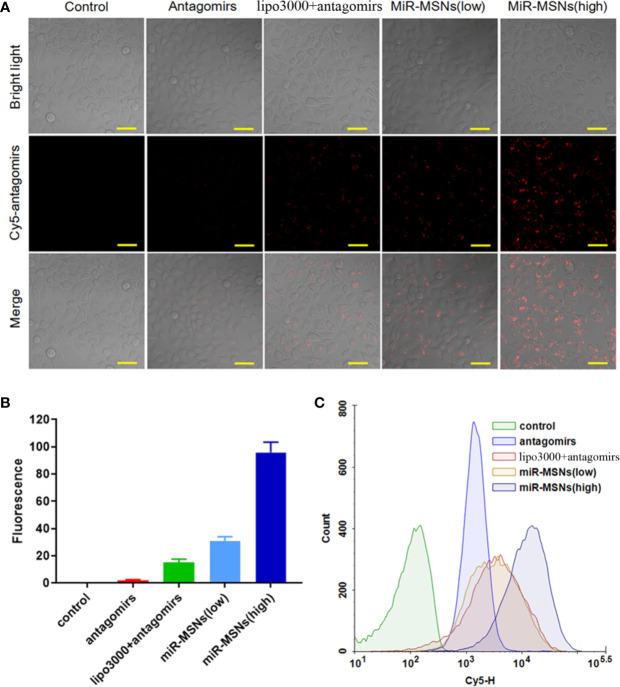
Intracellular uptake analysis. **(A)** CLSM images of cells after incubation with different Cy5-antagomirs formulations. Scale bar = 50 µm. n=3. **(B)** Corresponding quantitative results of CLSM images. **(C)** Flow cytometric histogram profiles of L02 cells after 3 h of incubation.

The extent of miR-33 antagomirs internalization in L02 cells was investigated by CLSM. L02 cells in the miR-MSNs (low) group were treated with 50 nM of miR-33 antagomirs while L02 cells in the other treatment groups were treated with equivalent miR-33 antagomirs (100nM). It can be observed that the fluorescence intensity of miR-33 antagomirs in L02 cells incubated with miR-MSNs for 3 h was higher than that of the cells in other groups (**Figure 2A**). From the statistical results, it can be inferred that miR-33 antagomirs in miR-MSNs exhibited approximately a 5-fold higher uptake in L02 cells when compared to lipo3000 in equivalent miR-33 antagomirs, thus confirming our hypothesis that NPs can enhance the endocytosis of miR-33 antagomirs to a much higher extent than lipo3000 (**Figure 2B**). The enhanced uptake of miR-33 antagomirs from miR-MSNs by L02 cells was further demonstrated by flow cytometry (**Figure 2C**); these results also correspond with the results of CLSM analysis. The enhanced intracellular uptake of miR-33 antagomirs by miR-MSNs may be attributed to the distinct cellular internalization of MSNs; they can be taken up by cells *via* various routes, such as caveolae-mediated andclathrin-mediated pathways (Zhou et al., 2018).

### *In Vitro* Regulation Effects of Lipid Metabolism

**Figure 3:**
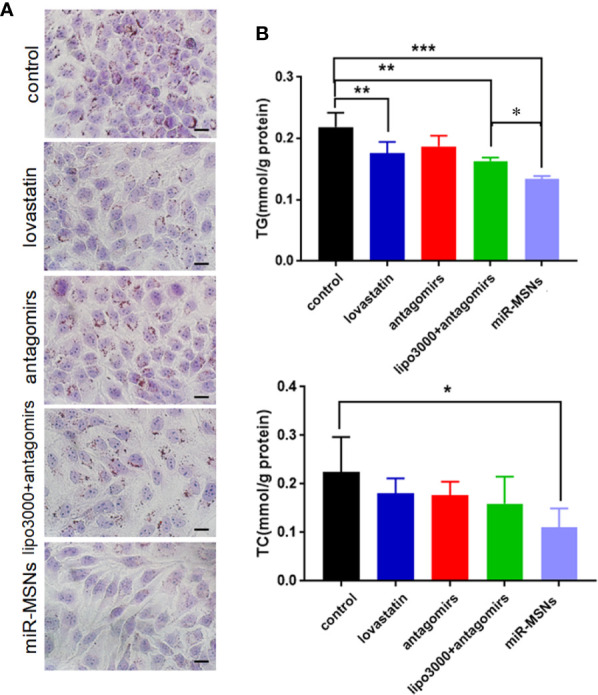
Anti-steatosis activity of miR-MSNs in L02 cells. **(A)** Determination of lipid accumulation using Oil Red O. Scale bar = 20 µm. **(B)** TG and TC levels in the cells. n=3. *p < 0.05, **p < 0.01, and ***p < 0.001.

To further define the effects of miR-MSNs on lipid accumulation, we applied the well-established *in vitro* model of lipid accumulation (OA administration model). L02 cells were treated with lovastatin, miR-33 antagomirs, miR-33 antagomirs+lipo3000, or miR-MSNs and then incubated with OA for 72 h. Lovastatin, a common clinical drug for dyslipidemia, was considered as the positive control (Downs et al., 1998). The Oil Red results showed that miR-MSNs significantly inhibited lipid accumulation in L02 cells when compared to the untreated control group and other treated groups (**Figure 3A**). Furthermore, we examined TG and TC levels in L02 cells. TG levels in the miR-MSNs group was significantly lower than that in the control group and other treatment groups. Meanwhile, TC levels in the miR-MSNs group was much lower than that in the control group, but there were no significant differences between other treatment groups and the control group, which indicated that miR-MSNs exhibited the best lipid-lowering effect among all the tested groups (**Figure 3B**).

### *In Vitro* Transfection Efficiency and Altered Gene Expression

**Figure 4:**
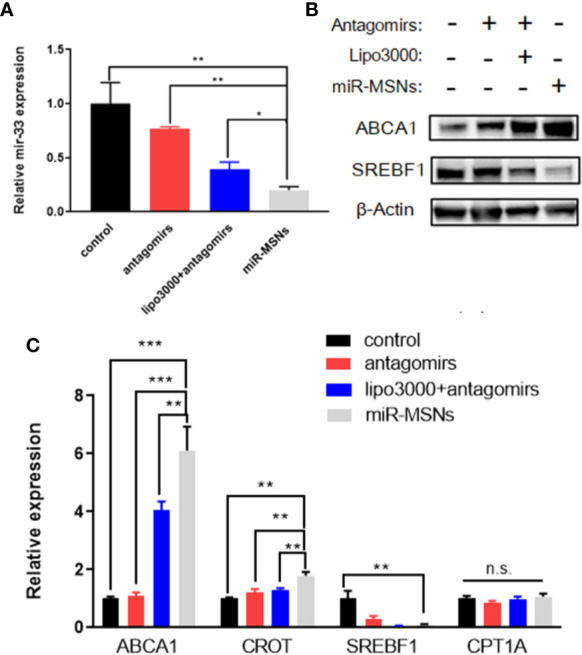
Silencing effect of miR-33 by miR-MSNs and changes in the expression of downstream genes. **(A)** Expression of miR-33 quantified by RT-PCR. n=3. **(B)** Western blot analysis of ABCA1 and SREBF1 protein. **(C)** Expression of miR-33 downstream genes (ABCA1, CROT, SREBF1, and CPT1A) quantified by RT-PCR. n=3. *p < 0.05, **p < 0.01, ***p < 0.001. n.s., no significance.

To determine the transfection efficiency of miR-33 antagomirs in miR-MSNs using an *in vitro* lipid accumulation model, L02 cells were treated with miR-33 antagomirs, miR-33 antagomirs+lipo3000, or miR-MSNs. We found a greater reduction of miR-33 in miR-MSNs–treated cells when compared to other groups (**Figure 4A**). We also observed changes in the mRNA levels of miR-33 downstream genes involved in lipid metabolism (ABCA1, CROT, SREBF1, and CPT1A). As shown in **Figure 4C**, the mRNA expression of ABCA1 and CROT was higher than that of other groups, while that of SREBF1 was lower than that of the control group. These results are consistent with previously reported results (Rayner et al., 2010; Rayner et al., 2011a; Rayner et al., 2011b). Meanwhile, the mRNA expression of CPT1A did not change after incubation, which is not consistent with *in vivo* observations of the above research. However, other studies reported that there are no marked changes in the expression of CPT1A in HepG2 cells and Huh7 cells after antagomir 33 transfection (Gerin et al., 2010; Goedeke et al., 2013). Hence, it might be inferred that the effect of anti-miR33 on CPT1A is different between cells and animals; this aspect requires further investigation. In addition, as miRNAs can affect both mRNA stability and translation, we measured two main functional protein levels (ABCA1 and SREBF1) in L02 cells. As shown in **Figure 4B**, ABCA1 was present at much higher levels in cells treated with miR-MSNs when compared to cells in other groups; meanwhile, the opposite trend was observed for SREBF1. These changes in protein expression influence lipid metabolism in a manner consistent with previous results.

### *In Vivo* Biodistribution Study

**Figure 5:**
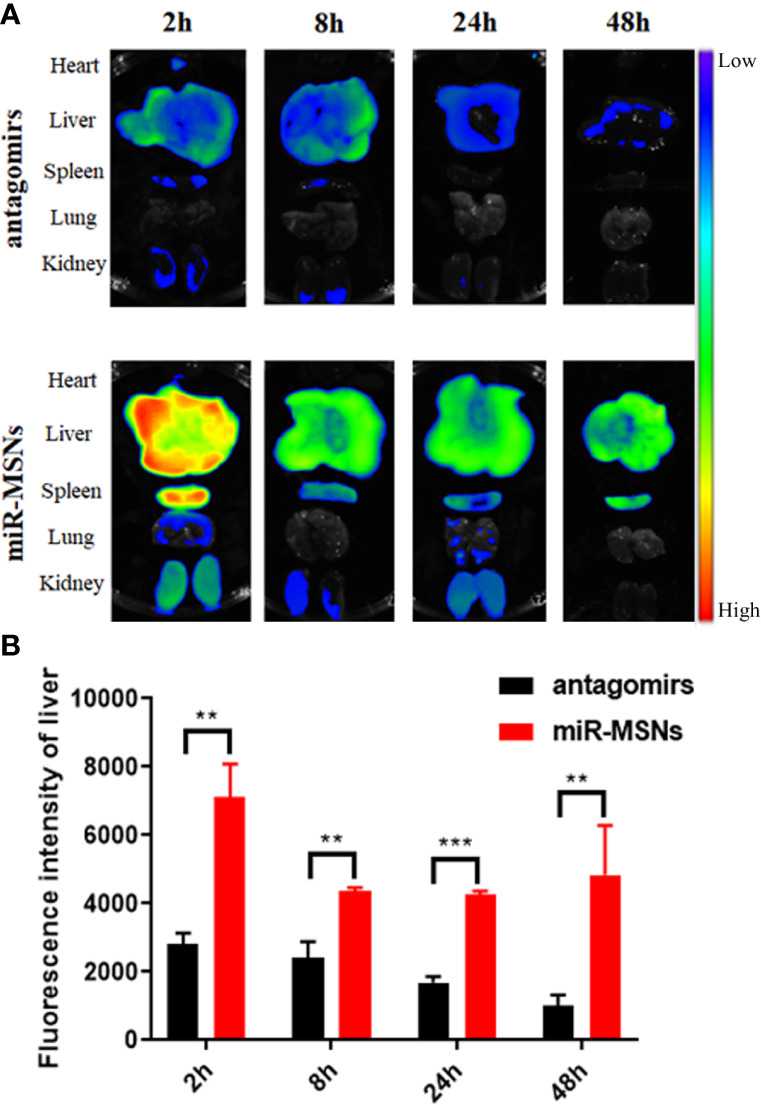
Bio-distribution evaluation. n=3. **(A)** Ex vivo fluorescence images of major organs (heart, liver, spleen, lung, and kidney) after administration. **(B)** Quantitative analysis of the distribution of miR-33 antagomirs in the liver. **p < 0.01, and ***p < 0.001.

To evaluate the *in vivo* distribution of miR-33 antagomirs and delivery capacity of miR-MSNs, mice were intravenously injected with miR-MSNs *via* their tails. The distribution of miR-33 antagomirs can be tracked by ex vivo fluorescence imaging of Cy5-labled miR-33 antagomirs in different organs. As shown in **Figure 5A**, a strong fluorescence signal could be detected in liver tissues for two groups 2 h after injection. The liver fluorescence intensity of the antagomirs group gradually decreased along with time, while that of the miR-MSNs group still maintained a high level 48h after injection. Moreover, the liver fluorescence intensity of the miR-MSNs group was observed to be significantly stronger than that of the antagomirs group at each predetermined time point (**Figure 5B**). These results suggest that miR-MSNs could protect miR-33 antagomirs and prolong their retention time *in vivo*; thus, a large dosage of miR-33 antagomirs produces a good therapeutic effect. miR-MSNs are also mainly distributed in the liver and spleen, which is similar to that of MSNs (He et al., 2011).

### *In Vivo* Regulation Effects of Lipid Metabolism

**Figure 6:**
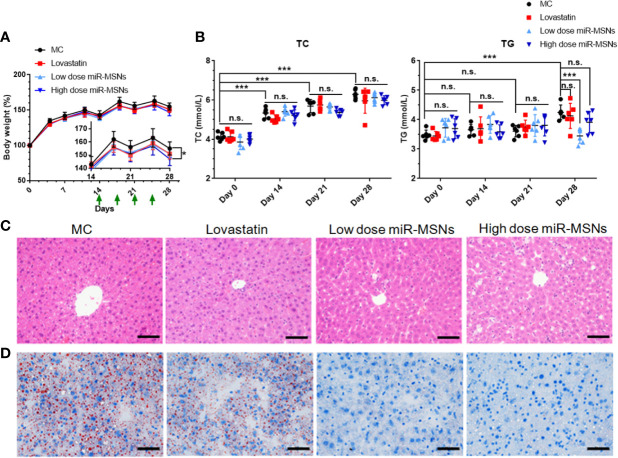
Lipid-metabolism regulation efficacy of miR-MSNs. n=6. **(A)** Weight change curve of HFD-fed C57BL/6J during the course of the study. **(B)** Concentration of TC and TG in the serum. **(C)** Representative photographs of HE-stained liver sections. Scale bar = 50 µm. **(D)** Representative photographs of Oil Red O stained liver sections. Scale bar = 50 µm. *p < 0.05, ***p <0.001. n.s., no significance.

We used HFD-fed mice models to assess the *in vivo* effects of miR-MSNs. Mice fed on HFD for two weeks were divided into four groups – MC, lovastatin, low dose miR-MSNs, and high dose miR-MSNs groups – and they were injected with PBS, 5 mg/kg lovastatin, 25 mg/kg miR-MSNs, and 50 mg/kg miR-MSNs twice a week *via* their tails. These mice were then sacrificed after 4 weeks. The mice in all four groups gained considerable body weight over the course of the study. However, the body weight of the high dose miR-MSNs group decreased significantly when compared to the MC group at Day 28. The body weight of the lovastatin and low dose miR-MSNs groups was reduced to some extent when compared to the MC group at Day 28, while no significant change was observed (**Figure 6A**). The reason for this may be the inadequate observation time; other studies reported a marked weight difference in animal models over two months of observation (Guo et al., 2019; Zai et al., 2019). To evaluate the effect of the synthesized NPs on dyslipidemia, TC and TG levels were measured on Day 0, 14, 21, and 28 (**Figure 6B**). TC and TG levels increased steadily in the MC group, indicating that our model was successful. On Day 28, TG levels in the low dose miR-MSNs group decreased significantly when compared to MC group and lovastatin groups, thus demonstrating the excellent effect of miR-MSNs on TG regulation in the serum when compared to the commercial drug. However, TC levels increased in all groups over the course of the study and there were no significant differences between the four groups at any given time point. ABCA1 is one of the main target proteins of miR-33 and its function is critical to the biogenesis of high density lipoprotein (HDL) and the efflux of excess cholesterol in the liver (Cohen et al., 2004; Wang et al., 2004). In serum, HDL increases with interference from miR-33 (Rayner et al., 2011a; Rayner et al., 2011b). There is strong evidence that high levels of circulating HDL are associated with positive cardiovascular outcomes (Viseshakul et al., 1979; Wilson Peter et al., 1998). Therefore, the increase in HDL, a component of total serum cholesterol, in miR-MSNs-treated mice may influence the change of TC levels.

Furthermore, hepatic steatosis in the tested mice was analyzed using HE and Oil Red staining of liver tissues (**Figures 6C, D**). While the lovastatin group exhibited reduced hepatic steatosis compared to the MC group, the low dose miR-MSNs and high dose miR-MSNs groups exhibited drastically reduced lipid accumulation in the liver. In fact, on Day 28, the lipid accumulation in these groups was similar to that of the pre-treated liver on Day14. These results illustrate the excellent therapeutic effect of miR-MSNs on NAFLD and dyslipidemia.

### *In Vivo* Safety Study

**Figure 7:**
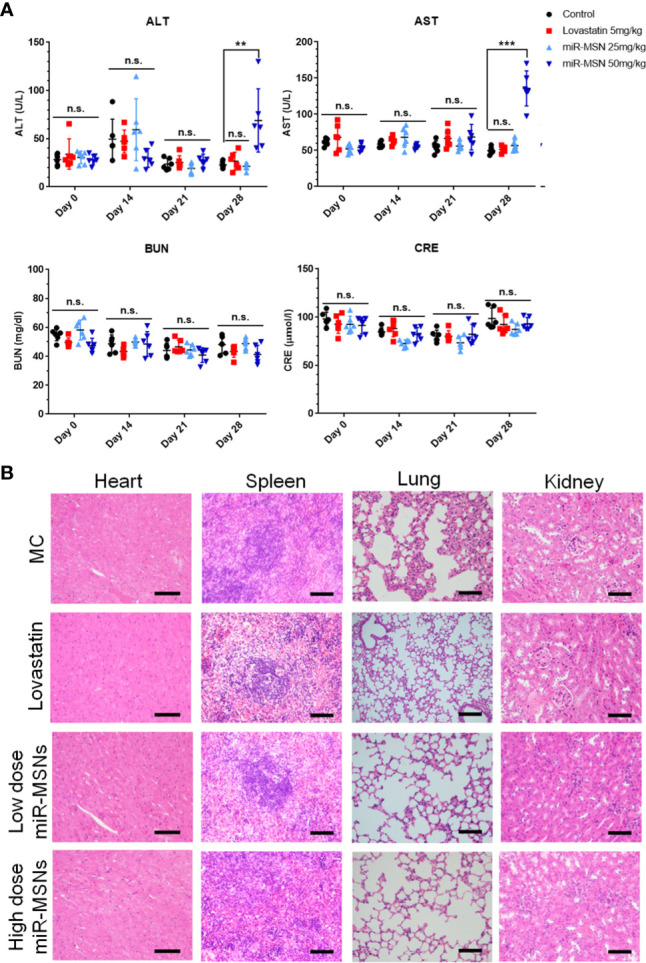
Safety analysis. n=6. **(A)** Concentration of AST, ALT, BUN, and CRE in serum on Day28; **(B)** Representative tissue sections of mice stained with HE on Day28. Scale bar = 50 µm. **p < 0.01, ***p < 0.001. n.s., no significance.

The safety of the NPs designed in this study was investigated in terms of their biocompatibility. As shown in **Figure 7**, there were no obvious histological differences in the major organs of treated and untreated mice. Except for the high dose miR-MSNs group, there were no significant differences between the treated groups and untreated group in terms of their ALT, AST, BUN, and CRE plasma levels, indicating the good tissue compatibility of the synthesized miR-MSNs. However, the rise in ALT and AST plasma levels in the high dose miR-MSNs group indicates potential liver toxicity. This finding is consistent with a previous report (Liu et al., 2011). To reduce liver toxicity, we recommend a NP dosage of 25 mg/kg for animal experiments; at this level, a similar therapeutic effect was observed. However, studies should be undertaken on the long-term *in vivo* toxicity of these NPs.

## Conclusion

NAFLD and dyslipidemia seriously affect public health, especially in LT recipients, who are at a high risk of metabolic diseases. These lipid metabolic disorders not only reduce patients’ quality of life, but also adversely affect long-term survival. Several lipid-lowering drugs are often used in clinical settings to treat dyslipidemia. However, these drugs are not useful for treating NAFLD. Statins are one of the most common lipid-lowering drugs used for patients. Some trials even reported the prevalence of NAFLD was higher in a group with statins (Sigler et al., 2018). Therefore, in this study, we attempted to develop a therapeutic nano-system superior to statins for both NAFLD and dyslipidemia.

We successfully synthesized a novel system of miR-MSNs for delivering miR-33 antagomirs to the liver. In vitro cellular uptake assay of miR-MSN NPs, which exhibited a hydrodynamic size of ∼120 nm, indicated that they entered hepatocytes specifically at a much higher efficiency than lipo3000. In vitro and *in vivo* experiments demonstrated that affected lipid metabolism, to a greater extent than lovastatin, were biocompatible and essentially nontoxic. Thus, we could conclusively prove that MSNs as delivery vehicles for miR-33 antagomirs represent a promising gene therapy system for lipid metabolic disorders. Further studies are needed to modify MSNs with hepatic-targeting ligands, such as lactobionic acid and hyaluronic acid, for enhancing the accumulation of NPs in liver.

## Data Availability Statement

The raw data supporting the conclusions of this article will be made available by the authors, without undue reservation, to any qualified researcher.

## Ethics Statement

The animal study was reviewed and approved by the Institutional Animal Care and Use Committee of Zhejiang University.

## Author Contributions

Conceptualization, LX and XX. Investigation, YT and SX. Methodology, YT and SX. Resources, YT and CZ. Writing—original draft preparation, YT. Writing—review and editing, all authors. Visualization, YT and SX. Supervision, XX. Project administration, JW. Funding acquisition, XX and JW.

## Funding

This work was supported by the National Natural Science Foundation of China (81801824, 81930016), China National Funds for Distinguished Young Scientists (81625003), National Major Science and Technology Projects of China (No. 2017ZX10203205), and Key Research & Development Plan of Zhejiang Province (No. 2019C03050).

## Conflict of Interest

The authors declare that the research was conducted in the absence of any commercial or financial relationships that could be construed as a potential conflict of interest.
