# Supplementary figures and images for: Delivery of microRNA-33 Antagomirs by Mesoporous Silica Nanoparticles to Ameliorate Lipid Metabolic Disorders
Source: Front Pharmacol. 2020 Aug 5;11:921. doi: 10.3389/fphar.2020.00921 (PMC7419650; doi:10.3389/fphar.2020.00921)

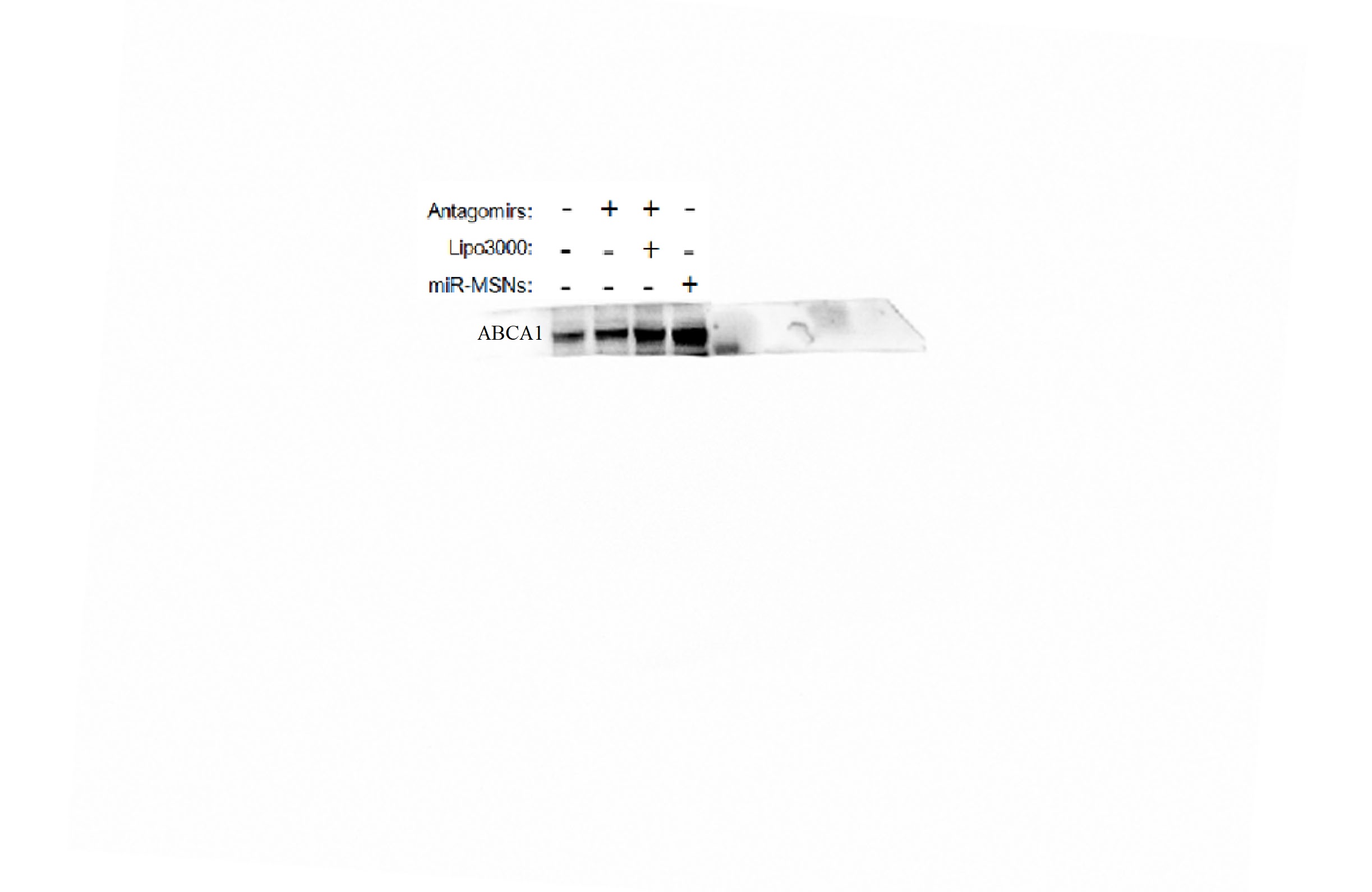

Supplement: Supplementary file 1 [file DataSheet_1.zip › ABCA1.jpg]

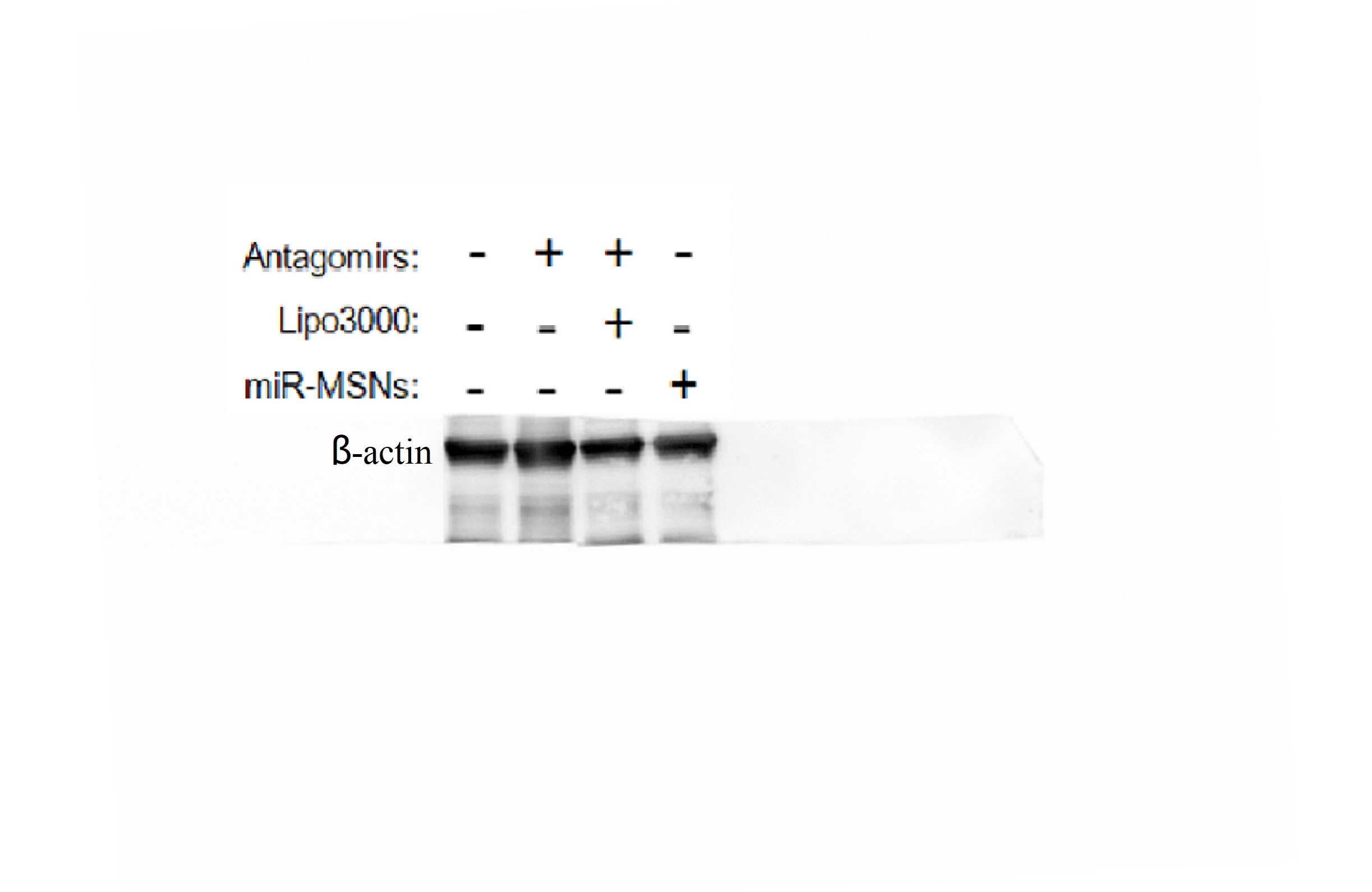

Supplement: Supplementary file 1 [file DataSheet_1.zip › actin.jpg]

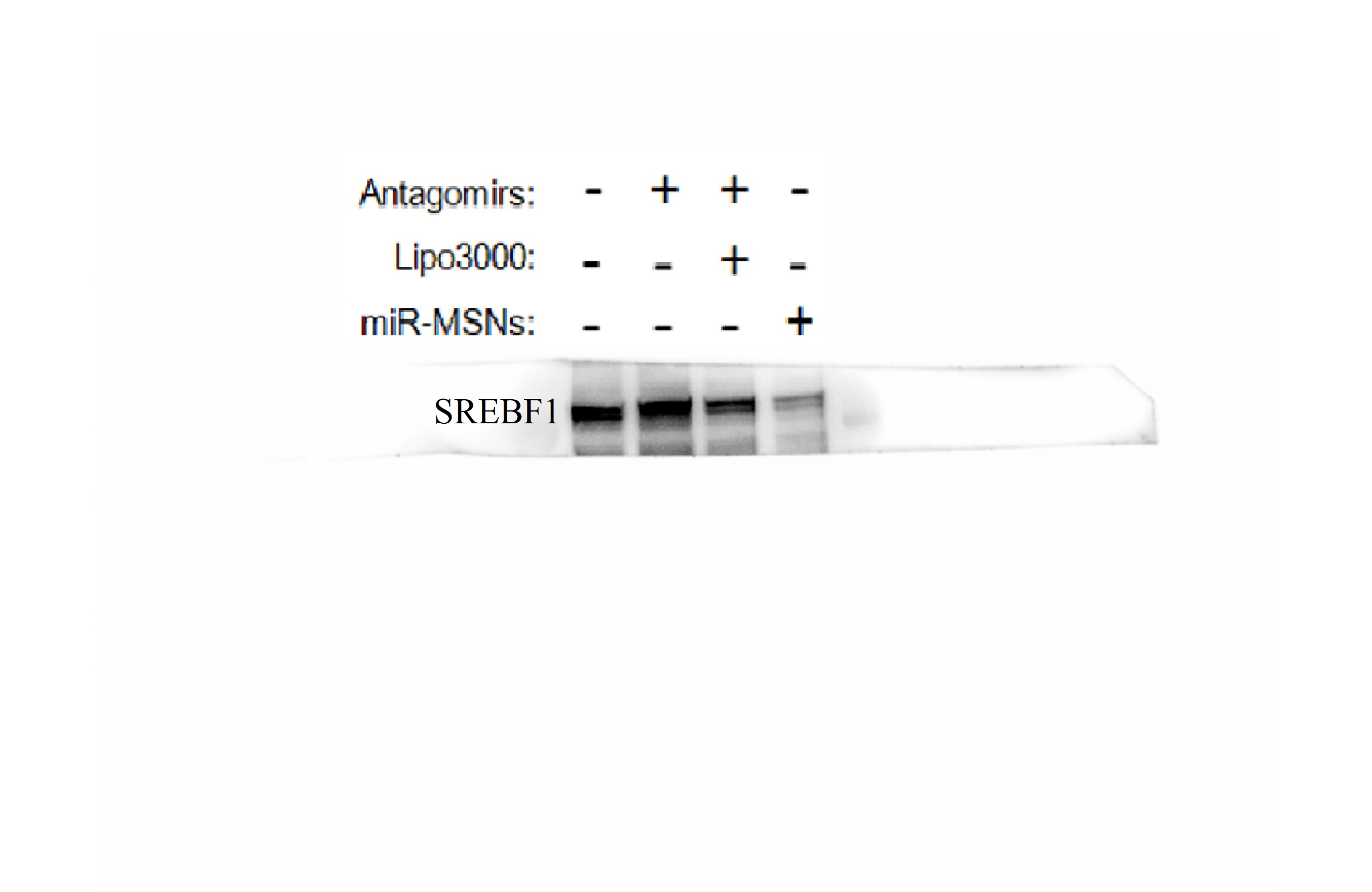

Supplement: Supplementary file 1 [file DataSheet_1.zip › SREBF1.jpg]
