# Supplementary material for: Delivery of microRNA-33 Antagomirs by Mesoporous Silica Nanoparticles to Ameliorate Lipid Metabolic Disorders
Source: Front Pharmacol. 2020 Aug 5;11:921. doi: 10.3389/fphar.2020.00921 (PMC7419650; doi:10.3389/fphar.2020.00921)
Supplement: Supplementary file 2 [file DataSheet_2.docx]

Supplementary Material

## Supplementary Figures

**
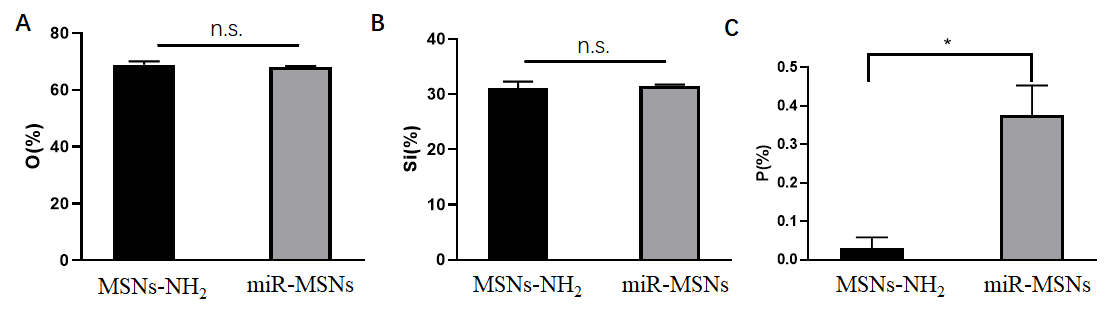
 Supplementary Figure 1.** X-ray energy dispersive spectrum (EDS) of MSNs-NH_2_ and miR-MSNs. (A) The proportion of O element. (B) The proportion of Si element. (C) The proportion of P element.


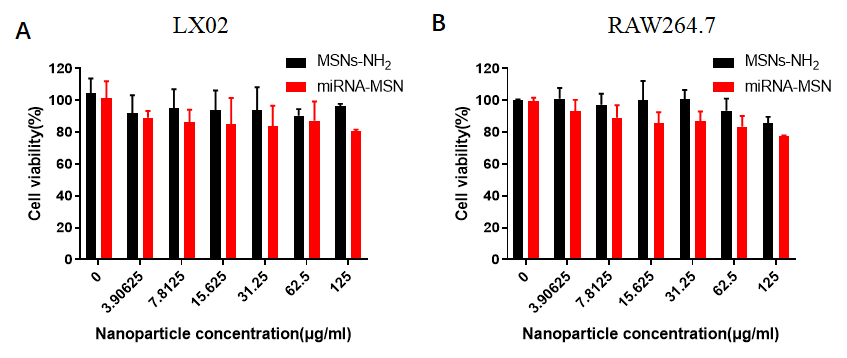


**Supplementary Figure 2.** Cytotoxicity of MSNs-NH_2_ and miR-MSNs in LX02 (A) and RAW264.7 (B) cell lines.
